# Acts of negotiation: toward a grounded theory of nursing practice in chronic wound care in Austria

**DOI:** 10.1186/s12913-023-10276-2

**Published:** 2023-11-14

**Authors:** Deborah Drgac, Raffael Himmelsbach

**Affiliations:** 1 Department of Political Science, University of Vienna, Universitätsstraße 7/2, Vienna, 1010 Austria; 2 Research Group Senescence and Healing of Wounds, Ludwig Boltzmann Gesellschaft, Donaueschingenstraße 13, Vienna, 1200 Austria

**Keywords:** Chronic wound care, Interprofessional collaboration, Therapeutic relationship, Experiential knowledge, Austria

## Abstract

**Background:**

Demographic change and the rise of diabetes mellitus are leading to a projected increase in the prevalence of chronic wounds. People suffering from chronic wounds experience significant losses in their health-related quality of life. Health systems struggle to meet the needs of these persons, even in high-income countries. This paper explores wound nurses’ perspectives on their professional practice in Austria. They play a key role as they do much of the treatment work, contribute to advancing the field, and enable interprofessional coordination. Their perspectives enable insights into how a health system provides care for elderly and chronically ill people.

**Methods:**

We used the Constructivist Grounded Theory framework to analyse transcripts of 14 semi-structured qualitative interviews with nurses who work in different treatment settings.

**Results:**

We identified three themes. Firstly, the interviewees characterise working with patients as a balancing act between offering enough support to build a trustful relationship while protecting themselves against the overwhelming situation of caring for a chronically ill person. Secondly, the interviewees compensate for nonexistent care pathways by building informal networks with doctors, which requires delicate relationship work. Thirdly, the study participants must prove their competence in every new professional encounter. Their need for professional autonomy clashes with the traditional doctor-nurse hierarchy. Based on these insights, we propose a grounded theory that conceives of nursing practice in terms of ‘acts of negotiations’.

**Conclusion:**

Our results demonstrate that wound nurses in Austria operate in an institutional environment whose outdated imagination of the nursing role is at odds with the care demands that arise from a growing number of elderly and chronically ill people. We detailed the ‘acts of negotiation’ nurses deploy to compensate for this situation. We identify areas for policy intervention to strengthen the autonomy of wound nurses, including access to statutory health insurance billing.

**Supplementary Information:**

The online version contains supplementary material available at 10.1186/s12913-023-10276-2.

## Introduction

This study aims to contribute to an empirically grounded theory of nursing practice in chronic wound care. Chronic wounds pose a major challenge for health systems as a growing patient population with complex care needs meets a stagnant health workforce [1]. Chronic wounds are skin lesions with protracted or no healing tendencies, despite professional medical treatment [2]. The most prevalent causes are vascular and metabolic diseases that contribute to tissue damage, typically in the lower leg or foot. Infection, poor nutrition, smoking and stress can further impair the healing of all types of wounds, though chronicity is uncommon in otherwise healthy individuals [3]. The disease prevalence is estimated to be 2.21 per 1000 population [4], with a rising trend due to demographic ageing and an increase in the incidence of diabetes mellitus [5]. Patients require weekly, if not more frequent medical treatment sessions that take 15 min or more, and typically involve a dressing change. Moreover, they require treatment over extended time periods: healing times for venous leg ulcers, for instance, are estimated between 1 and 5 years [6]. Even when wound closure has been achieved, secondary prevention measures are necessary to avoid the formation of a new wound [6–8]. This adds up to a significant demand for professional care time, which is set to increase with the rising disease prevalence.

It is predominantly nurses who perform wound care and it is also they who are impacted by the care situation. The impact of chronic wounds on patients’ lives is significant. They experience pain, sleeping difficulties, loss of mobility, social isolation, and poor mental health as a result of their wounded condition [9]. Previous studies on chronic wound management have found that nurses invest in the quality of therapeutic relationships, which they see as exerting a curative effect on the patient and as constituting a rewarding situation for themselves. These studies also show that therapeutic relationships can be overwhelming for nurses, and lead to a sense of helplessness when wounds do not heal and the physical, emotional, and social distress of the patient cannot be alleviated [8, 10]. This sense of impotency can negatively affect the therapeutic relationship and undermine treatment progress [10].

Beyond the therapeutic relationship, we know rather little about nursing practice in chronic wound care and what shapes it. Wound nurses are said to be predisposed to a coordinating role between different health services, due to the emergence of this nursing specialisation outside the confines of a single medical discipline [11]. Moreover, there is recognition that a multidisciplinary team approach improves patient outcomes [12]. This implies coordinated communication and action across professions, and a treatment setting that includes the patient as well as lay caregivers. Nurses likely assume the coordinating role necessary to operate as a team. A team approach is a departure from the traditional doctor-nurse hierarchy where doctors diagnose and delegate care to nurses. In a team setting, nurses gain autonomy, which in turn implies doctor-to-nurse task shifts. The experience thus far with implementing advanced practice nurse roles in various European countries, a case of task shift in practice, shows that this implies systemic and cultural changes that are not equally embraced by all professions [13, 14].

Taken together, the projected increase in the demand for nursing care for chronic wound patients [5], a potentially demanding therapeutic relationship [8, 10], and changing professional relations [13] are three important reasons for investigating nursing practice in this domain. The question guiding our study thus becomes: How do nurses experience their care work with chronic wound patients and what does it mean to them? Nurses’ perspectives on their practices, concerns and meaning-making allow us to identify those aspects of care situations that they experience as consequential for their care work with patients.

For this purpose, we conducted 14 semi-structured interviews with wound nurses working in different healthcare settings in Austria. We use Austria as a case because, overall, it has a high-performing and expensive healthcare system with near-universal social health insurance coverage [15] and a well-established long-term care system [16]. But when zooming in on chronic conditions, ‘invisible barriers’ [17] and a lack of scaled-up chronic disease management programs [18] become apparent, raising questions about the system’s performance in that regard. Serving chronic wound patients with a coordinated health service approach appears to be a challenge for health systems more broadly and may require a dedicated national programme, as is for instance the case in the UK [19]. To date, the Austrian health system lacks a coordinated approach to chronic wounds. Instruments like quality standards and treatment algorithms are not uniform and are even completely missing in some parts of Austria. Moreover, there is widespread discontent among wound professionals with this *status quo* [20].

This paper is structured as follows: the next section provides background on chronic wound care in the Austrian health system. The methods section details the research design of this qualitative interview study and explains our use of Constructivist Grounded Theory for data analysis. The results section presents the thematic codes that resulted from the analysis. We then discuss these findings and suggest a theory of nursing practice in wound care on this basis. In the conclusion, we argue the relevance of our findings and offer policy implications.

## Chronic wound care and the Austrian health system

Care fragmentation in Austria is a well-known fact. It is caused by a complex governance system due to federalism and the delegation of some governance responsibilities to non-state actors, such as the doctors’ chamber and statutory health insurance entities. The primary care sector is nominally responsible for patients with chronic conditions. There is a small number of disease-specific integrated care pathways that seek to address care discontinuities between the different care settings [18]. No such pathway exists for chronic wounds. There is evidence of informal multidisciplinary networks that community care nurses try to sustain to compensate for the lack of formal structures. These arrangements are complicated for nurses, as they are not on the same institutional footing as the doctors on which they depend [21]. Unlike physiotherapists, for instance, the services of registered nurses who are self-employed are not eligible for reimbursement by the statutory health insurance, though they can invoice private clients [21].

The professionalisation of wound care nursing is relatively recent in Austria. It takes place between the bottom-up self-organisation of practitioners and the formal system of nurse education. The former precedes the latter and has a continuing parallel existence. Voluntary associations for wound care professionals form its backbone. They organise conferences and social activities. Like in the UK, this community co-exists in symbiosis with the medical device industry [22], with an industry-led policy platform providing sponsorship for events and organisational capacity for lobbying [20].

The system of nursing education is in transition from vocational to academic training. Bachelor’s degrees were first introduced in 2016. The same reform saw the introduction of a list of continued education specialisations, ‘wound management’ being one of them. The caveat of this reform is that it only labelled but not defined ‘wound management’, neither in terms of tasks nor number of training hours. By now, several different types of certificates are offered by academic and non-academic providers. Notwithstanding the lack of clarity about the profile of wound management, it is now being used for the purposes of health workforce planning [20]. National statistics show that about 2 percent of the national nursing workforce have obtained one of the various wound management certificates; just under one-third of them are listed as self-employed [23]. The professionalisation of wound nursing thus seems to be an evolutionary process whose fate will depend on the larger question of nurse education.

## Methods

### Methodological framework

This study is part of a larger project that investigates different aspects of chronic wound care in Austria. The data used for this study consists of 14 semi-structured qualitative interviews that we conducted with nurses who specialise in wound treatment across a broad range of care settings. We employed the Constructivist Grounded Theory (CGT) [24] framework for developing an empirically situated theory of nursing practice in chronic wound care in Austria. It is widely used in the nursing and health sociology research fields [25].

### Constructivist Grounded Theory principles

The CGT provides a protocol for theorising based on qualitative (interview) data. It allowed us to examine how wound nurses experience their work, the interpersonal relationships that are part of it, and how more abstract concepts, such as education and law, figure in their lifeworlds.

The principles of credibility, resonance, originality, and usefulness are used as quality criteria in the CGT [26]. The credibility of our analysis is demonstrated by its clear aim and transparent theoretical sampling of interview participants, which was stopped once thematic saturation was achieved. To guard against the researchers' assumptions unduly influencing the analysis, we worked as a co-author team. In addition, Drgac regularly met with a peer group unrelated to the present project for data analysis sessions. We checked for the resonance of our findings with perspectives that were similar, but not identical to our interviewees’ through participating in wound conferences and by presenting parts of the findings in co-creation workshops with health professionals that we co-organised as part of the larger project this analysis is part of. The originality of our study derives from taking seriously the (Austrian) health system as the context of nursing practice. We consider our results useful because they highlight burdens placed on nurses that negatively impact care situations. Moreover, we offer policy recommendations as part of the conclusion.

### Analytic procedure

#### From research question to theory building

**Fig. 1:**
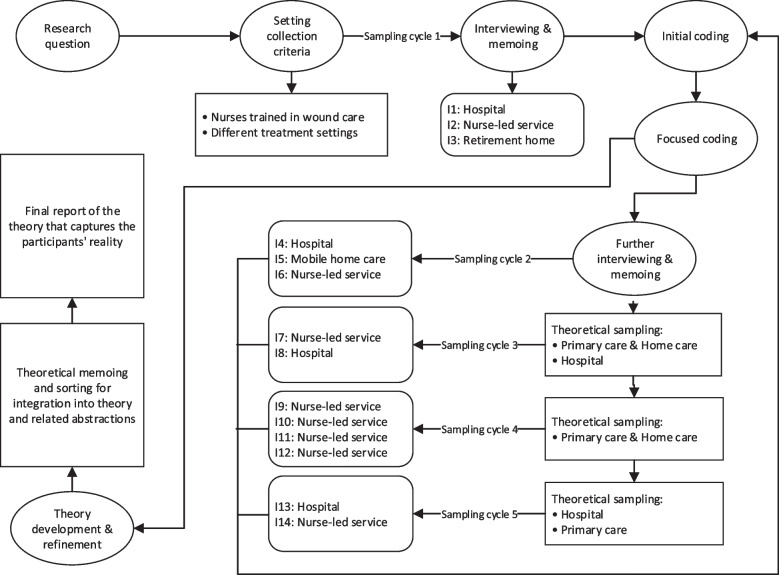
The CGT research process. Own illustration adapted from Lauridsen et al. [27]

Figure 1 illustrates our analytical strategy, which we adapted from Lauridsen et al. [27]. Having defined our research question, we established criteria for generating qualitative interview data (see below). After conducting an initial set of interviews, we started coding the transcripts. Based on the evaluation of the repetition and coherence of the codes, we could then determine if further interviews were needed. This procedure was repeated five times. We compared new data with previously collected information at each iteration to identify new patterns and validate those previously established. We repeated this iterative process of data collection, analysis, coding and categorising, as well as theory refinement until we reached a point where no new categories emerged. At this stage, we reached a point of saturation where additional data would not contribute further insights to the development of our theory. It is at this juncture of saturation that our theory of nursing practice has been fully constructed.

#### The interview guide

Our interview guide was designed to lead the conversation from concrete, everyday topics of nursing practice to more abstract topics that were not part of the participants’ everyday professional lives. We started the interviews by asking about the participants’ daily professional tasks; we then moved on to their interactions with patients and family carers. We subsequently delved into interprofessional relationships that are relevant to patient care. We ended by eliciting the interviewees’ experiences with professional education and working in the Austrian healthcare system more broadly. We posed open-ended questions that we adapted to each interviewee’s situation and followed up where we wished for more detail [28]. The iterative approach inherent in theoretical sampling also meant that we introduced additional questions to the topic guide as they arose from the previous analysis cycle. The interview guide can be viewed under the Supplementary files.

#### Recruiting interview participants

The interviews took place between August and December 2021. They lasted between 30 and 75 min. Due to pandemic-related contact restrictions, only online interviews were feasible. Participants provided written informed consent ahead of the interview. The interviews were held, transcribed, and analysed in German. Only the quotes used in this article were translated into English.

We followed a theoretical sampling strategy [29] with the only a priori restriction that we targeted registered nurses who specialise in wound care because it is the only health profession in Austria that specialises in this topic and presumably would offer the richest perspectives. Theoretical sampling means that interviewing and analysis are conducted in batches and that selecting whom to interview next depends on the topics that emerge through the data analysis. Our sampling strategy consisted of five cycles of interviewing and data analysis. Table 1 describes the interviewees.

**Table 1:** The final interview sample

Interview	Gender	Care setting	Patient co-payment
1	F	Hospital (in- and outpatient)	No
2	M	Nurse-led ambulatory wound service	Yes
3	M	Nursing home	No
4	F	Hospital (inpatient)	No
5	F	Mobile home care	No
6	M	Nurse-led ambulatory & home care wound service	Yes
7	F	Nurse-led ambulatory & home care wound service	Yes
8	F	Hospital (in- and outpatient)	No
9	F	Nurse-led home care wound service	Yes
10	F	Nurse-led ambulatory & home care wound service	Yes
11	F	Nurse-led ambulatory & home care wound service	Yes
12	M	Nurse-led ambulatory & home care wound service	Yes
13	F	Hospital (inpatient)	No
14	F	Nurse-led ambulatory & home care wound service	Yes

#### Analysis process

The data analysis process followed a sequence of steps to identify patterns in the interview transcripts. Firstly, we broke down the transcripts into manageable chunks; we examined them line by line and annotated their content. These annotations are called *initial codes*. They are phrased using active verbs to make clearly visible the actions and situations depicted in each line of the source data [24]. Secondly, we compared the initial codes to each other to identify emergent relationships and patterns. This resulted in *focused codes* [21]. In the final step, we considered the possibility of a *core category* that would offer a common logic behind the different focused codes [21]. One of the method’s peculiarities is that every transcript is reanalysed when a new initial code is added.

We wrote memos to document our evolving understanding of the data, connect concepts, spot patterns, and delve into category relationships during interpretation. Our memos were also used in data sessions with peers to foster the analysis’ credibility.

## Results

**Fig. 2:**
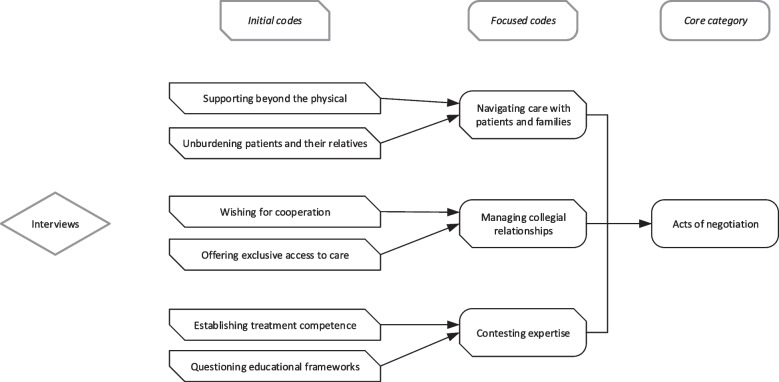
Concept map of empirical codes and their relationships

The analysis resulted in the identification of six initial codes, which in turn form the basis of three focused codes (Fig. 2). The results section describes these focused codes and their composite initial codes. We use verbatim quotes to illustrate particularly salient features of initial codes. They have been selected for their descriptive character but should not be read as an exhaustive summary of the underlying initial code. The presentation starts with the focused code “navigating care with patients and families”, followed by “managing collegial relationships”, and ends with “contesting expertise”. In the subsequent discussion section, we shall argue that the pattern of perceived social reality expressed in the focused codes share the common core category “acts of negotiations”, which we consider at the heart of a grounded theory of nursing practice in chronic wound care.

### Navigating care with patients and families

Wound treatment involves substantial contact time between nurses and patients, which they spend in hospitals, surgeries, and patients’ homes. The following presents topics related to this contact time solely between nurses and patients that emerged from the data analysis.

#### Supporting beyond the physical

Many interview participants described that they had established relationships of trust with their patients that sometimes exceeded what they considered a ‘normal therapeutic relationship’. The interviewees reported noticing that fostering a strong bond had a positive psychological effect on their patients. This connection was also observed to impact wound healing beneficially. "*[The wound] was in a way mirroring [the patient’s] soul*" (I7), an interviewee explained.

According to the interviewees, there is ‘a social component’ in wound care of therapeutic significance, especially in the context of people being treated at home. An interviewee described an elderly patient who spends more time in direct contact with her nurse than her family, who allegedly only visit once per week for 30 min. What might be a stereotypical situation of old age also applies to younger patients in the care of our interviewees. A wound nurse explained that young patients are prone to feeling ashamed of their wounds as they think it is a condition that is rather typical for ‘old people’. He further maintained that the impact on one’s life is rather dramatic when a wound interferes with the ability to hold a job due to pain or exudates.

Besides the comforting effect of a close relationship, some interviewees also aimed to build a strong personal connection to understand the social setting in which their patients are situated. Such knowledge helped the wound nurses to determine what resources a patient had that could be activated to increase well-being. Interviewees relayed their experience that repeated contact between the same patient and carer makes it more likely that a patient follows health advice concerning nutrition and physical activity, increasing the probability of faster wound healing.

The psychological burden that came with a chronic wound was mentioned in several interviews. A recurrent trope is that the treatment of the physical wound is simultaneously a treatment of the soul. “*We see how the wound heals and how the patient is healing with the wound*” (I14). Some of the interviewed self-employed wound nurses shared that spending time with their patients also provides psychological support beyond what they consider possible in publicly funded hospitals or surgeries.

But having solid relationships with patients seemed to have downsides, too. Some wound nurses suspect individual patients manipulating their dressings to prolong treatment, thereby securing valuable social contact with their wound nurses. These patients allegedly extended their care dependency for more than two years in that way. Interviewees also described cases where patients shared their psychological issues with them but were unwilling to talk to a psychologist. The inconvenience of seeing another healthcare professional and the added financial burden were seen as reasons why wound nurses are used as one-stop-shop solutions by patients. (As discussed in more detail below, the services of self-employed nurses are an out-of-pocket health expenditure that represents a substantial financial burden to patients.) Moreover, wound nurses perceive patients as losing a sense of boundary, for ‘if my wound nurse treats this ugly part of my body at this intimate spot, I can tell them everything’. Some interviewees described being overwhelmed by the personal information given to them and felt not sufficiently qualified to provide advice and offer adequate psychological support to their patients.

#### Unburdening patients and their relatives

The interviewees emphasised that treating a chronic wound is a delicate undertaking. Wounds can get infected when handled in unhygienic conditions, leading to slower healing, oozing and malodour. Infected wounds can also spread germs to other parts of the body or other people. The delicacy of wound treatment was sometimes overwhelming for patients, who initially underestimated the required effort to treat their wounds. Several pitfalls were listed: Eager to assist, patients sometimes prepared dressing materials and opened the sterile packaging before the nurse’s arrival; meanwhile, the cat walked around the room, possibly touching the exposed, no longer sterile dressing material. Removing pets from the treatment room, airing sufficiently, and repeatedly changing gloves were considered basic steps that patients and relatives frequently forgot.

The following quote shows this:

It rarely happens that [wound treatment] is done by the family members because they are generally overwhelmed with the situation, the care of chronic wounds. Many cannot do it. (I1)

Several interviewees agreed with this statement. They were convinced that self-treatment or shared care didn’t pay off economically for patients; what they would save in the short term on nursing care, they would inevitably pay later in case of healing complications. Other interviewees, however, had more positive experiences of including relatives in a shared-care arrangement on condition that they were properly instructed on how to conduct a dressing change.

Negotiating shared care was about more than dressing changes. Our interviewees emphasised that patients and their family members were surprised and often overwhelmed by the number of tasks. For example, patients are responsible for procuring the dressing materials.

[The patients] then simply don’t have the dressings – they are not easily obtainable – or they lack the competency because they never learn that. (I2)

Patients and their relatives easily forget to check their stock of dressing material, or they might put off refilling a prescription because it would entail a GP consultation. When the wound nurse arrives for a home visit and the patient is out of stock, they are forced to improvise. Not having material corresponding to the treatment plan could lead to slower progression in wound healing, which was described as potentially frustrating for all parties.

Self-employed wound nurses reported that part of their service is managing their clients' procurement logistics. When doing so, they reported having better treatment conditions, as patients and their families were not responsible for ordering the suitable dressing material in the correct size. However, this service is not offered free of charge. Almost all interviewees pondered the financial burden of wound care for their patients. They pointed out that using a wound nurse’s service also means considering the ambivalence of better wound care on the one hand and high costs on the other. Several interviewees reported feeling guilty when their patients cannot pay, and family members must help financially. In some cases, the financial circumstances led family members to take over treatment partially. In those cases, a contract was drawn to protect the professionals from malpractice allegations.

### Managing collegial relationships

Our interviewees emphasised that a clear and consistently implemented treatment strategy is essential for effective wound treatment and for guiding patients through the sometimes lengthy experience. In primary and community care, a patient’s doctor and wound nurse often work for different organisations yet must cooperate to make a consistent treatment plan work. This could mean wound nurses must cultivate a professional relationship with a separate doctor for each patient. Nurse-doctor relationships must also be managed in a hospital setting, where physical proximity doesn’t necessarily equate to collaboration. The following section describes how wound professionals describe and manage these collegial relationships.

#### Wishing for cooperation

Our interviewees described the range of experiences they had in their professional interactions with their patients’ doctors. From a nurse’s point of view, a good relationship consists of a doctor following the nurse’s recommendation when it comes to dressing material prescriptions. Such a relationship was experienced as empowering and efficient as it backed the nurse’s treatment plan. A treatment plan must be understandable, and each step must be communicated to the patient. Patients would then feel safe, be able to express consent and be mentally prepared for the procedure.

However, nurses and doctors are only sometimes able (or interested) to find consensus on a treatment plan. A self-employed wound nurse recounted her experience of such a situation where she and the patient’s dermatologist offered contradictory advice.

Of course, the patient was completely confused because the dermatologist explained that what I was doing was utter nonsense and unnecessary and that she shouldn't believe me. But she trusted me and expressed that she feels a bit caught in the middle, not really knowing how to react, for all she wants is for the wound to heal. (I7)

Such situations were deemed confusing for patients and patronising for wound nurses. Alas, nurses cannot force doctors to consider their opinions. Some interviewees thought that having the right to prescribe would give them some leverage in such situations.

Being patronised was not the only grievance the interviewed nurses had. Another self-employed wound nurse reported regularly seeing patients who were feeling worse off after having their wounds treated by doctors without wound expertise. She describes the situation as follows:

Once they come to see me, they often have an arduous journey behind them, from various doctors to what I’d call regular home care nurses, before they are seen by wound specialists, and by then their suffering is rather substantial. But, that's the way it is, nobody consults me until the roof is on fire. (I11)

The interviewees describe a perceived mismatch between a patient’s default point of care in the health care system and the required expertise to successfully treat a chronic wound, which unnecessarily prolongs patient suffering. It is a common experience for interviewee 11 to hear from her patients that doctors removed dressings without evident medical cause, for instance, during a routine blood draw. She emphasised her willingness to assist with her expertise, offering photographic documentation of wounds and even direct consultation with doctors. However, her wish for cooperation was often one-sided.

A senior wound nurse at a hospital's cardiology unit described a similar scenario. The schedule of doctors’ rounds is often unpredictable, which makes it impossible to schedule dressing changes so that the wound is exposed in time for inspection by the attending physician. So, she changes dressings according to her schedule. But then, each visiting doctor would remove the dressing to inspect the wound, no matter how recently the bandage was changed. This can happen several times to a patient. The interviewee judged these repeated inspections to be unnecessary, painful for patients, prone to causing skin irritations, and detrimental to wound healing. Having to reapply the dressings repeatedly did not feel like her work was valued. A hospital care director pointed out that doctors' schedules take precedence over nursing work, irrespective of whether this makes therapeutic sense, the prevailing paradigm being ‘doctors order and nurses carry out’ and not deliberation among experts of different specialities.

More interviewees diagnosed a mismatch between wound nurses’ expertise and their simultaneous dependency on hierarchically superior but less competent doctors, which they considered to result in adverse outcomes for their patients. Two interviewees explicitly told us that doctors’ attitudes significantly influence whether and how a nurse’s expertise is considered.

When we asked our interviewees what they wished for, several answered they wanted more cooperation and argued that establishing more interdisciplinary networks would benefit comorbid patients. As a self-employed wound nurse complained: “*Professional groups work among themselves, no overlap is happening, there is no network philosophy whatsoever.*” (I13).

#### Offering exclusive access to care

Self-employed nurses must invoice their patients since statutory health insurance does not cover their services. Several interview participants belong to this category. They wish that the insurance covered their services. Two interviewees had independently sent inquiries to health insurance providers concerning possible coverage. Either they received no response, or the insurance stated in no uncertain terms that there was no requirement for wound nursing services to be covered in Austria. This revelation came as a significant shock to her and her colleagues.

Other wound professionals reframed the out-of-pocket status of their services as an opportunity to provide added benefits for their patients. Two interviewees claimed that their patients were more likely to be prioritised in hospitals and doctors’ surgeries due to the personal relationships the interviewees established with doctors and hospital staff. Two self-employed wound nurses reported visiting their patients’ family doctors and other surgeries to introduce themselves. They do this to ensure efficient access to care for their patients and to establish a direct line of doctor-nurse communication without the patient having to play the messenger between professionals.

While relationship building is seen as necessary, it is described as tricky.

You always must be excellent when you do patient visits in a retirement home. You must be better than any doctor because you must put yourself into the position to be accepted by the resident doctor as an expert in the wound domain. That’s important. (I2)

Several interviewees felt that establishing close relationships with doctors was an asset for mitigating the limited prerogative of the nursing profession.

### Contesting expertise

Doctors are hierarchically superior to nurses chiefly by their profession’s legal entitlements. The following elaborates on situations when legal status and expertise are not perceived to align.

#### Establishing treatment competence

The legal foundation of expertise in wound treatment was a recurrent theme in the interviews. As previously demonstrated, wound nurses judged the legal basis of nursing as curtailing their autonomy, with the right to prescribe as the touchstone of this grievance.

As reported by many, if nurses established a good relationship with physicians, doctors were willing to sign the prescription without seeing the patient. This was seen as a win–win scenario despite its murky legal basis. Our interviewees pointed out that physicians would benefit from this agreement because they could bill the insurance for issuing a prescription without a time-consuming consultation.

Some interviewees compared the Austrian legal framework with those from other countries, such as the UK, where tissue viability nurses are reportedly allowed to prescribe some dressing material and medication to treat a chronic wound.

I’m afraid the legal status in Austria is lagging, in my opinion, because nurses are not even allowed to prescribe therapeutic aids, which I find very unfortunate, let alone dressings, despite there being lots of expertise. And that should be used. It would be beneficial, also from the perspective of general practitioners. (I8).

Regardless of the treatment context, interviewed wound nurses admitted to performing acts that knowingly transgressed their legal competence, such as removing necrotic skin tissue. Our interviewees explained several reasons they do so: in some cases, patients wish to get the job done quickly rather than wait until a doctor has time to see them. Doctors seem to tacitly agree to nurses performing such interventions in the hospital setting if there is no medical emergency. Our interviewees spoke about those acts of transgression mainly to emphasise their point of view that the legal framework is too restrictive for daily practice and ought to be revised at a regulatory level.

Policy could empower the nursing sector […] and say, okay there are training programmes that are certified […] where the nursing sector is simply responsible … also concerning prescriptions and not just refills. (I15).

This interviewee pointed out that her specialisation as a wound nurse through continued education had no bearing on her professional autonomy, for she was but a registered nurse in the eye of the law.

#### Questioning educational frameworks

When our interviewees asked their patients how previous providers treated their wounds, they repeatedly got the same answer: disinfection sprays or iodine cremes. The interviewees emphasised that those products are inappropriate choices, too aggressive for already damaged skin and a tabula rasa approach to bacterial colonisation, which also kills off bacterial species beneficial to wound healing. The descriptions were similar regarding the dressing materials used, with dry dressings used indiscriminately of the wound type and state of healing.

An interviewee stated that neither nurses nor doctors receive sufficient wound treatment training as part of their education. For instance, doctors know too little about the diabetic foot despite its prevalence in clinical practice. He asserted that the training for doctors barely encompasses diabetic foot care. This led him to the concern that, as medical professionals embark on their careers, they are expected to manage diabetic foot prevention programmes without sufficient education.

Our interviewees were convinced that their wound expertise was superior to the average medical professional, enabling them to obtain better treatment outcomes. They thought of themselves as meeting the most recent standards of care. They often justified this claim by pointing to the continued education certificate of wound management that they obtained.

Although several interviewees argued that being certified wound nurses should enlarge the number of procedures they are allowed to perform, others perceived wound management training in Austria to be too heterogenous to serve as the legal basis for additional competencies. As this person pointed out:

What upsets me … whether people accomplish a basic course, or, like me, have done 18 modules, which took me four years, they are all allowed to call themselves wound managers. Everyone claims after two- or three-weeks training ‘I am a wound manager, and I am the best therapist’. There is no subdivision akin to nurse and nurse assistant […] people with only basic training are buzzing around on the market claiming to be the best and occasionally create havoc. (I6)

This interviewee thought that the vagueness of the term ‘wound manager’ is confusing to patients and potentially harmful to the reputation of the profession.

## Discussion

After having presented the three focused codes that emerged from the analysis – ‘navigating care with patients and families’, ‘managing collegial relationships’, and ‘contesting expertise’ – we now discuss them in relation to each other, the Austrian healthcare system, and the broader literature.

The prominence of the therapeutic relationship as a theme is hardly surprising, given how foundational it is to nursing work and how much time nurses spend with their wound patients, especially in the home care setting. Previous studies, not set in Austria, identified the same theme [8, 10]. Their findings are largely congruent with ours, from the hope invested in the curative effect of a trusting relationship, the holistic vision of care (“treating patients’ bodies and souls”), to nurses’ experiences of being overwhelmed by failing treatment and patients’ unfiltered emotional and physical pain. As Morgan et al. [10] argue, the latter can undermine a trustful therapeutic relationship when nurses blame patients, which they consider a coping mechanism, not ill intent. They single out the ‘social ulcer’ – the claim that some patients deliberately sabotage the treatment process to prolong social contact with caregivers – as a justification narrative. The patient’s suspect behaviour is never observed directly, the motive is inferred, and blame shifts responsibility from the nurse to the patient. We have encountered ‘social ulcer’ anecdotes not just in our interviews, but also at wound conferences in Austria and internationally. It is a story told from one professional to another. We suggest that the social ulcer is an element of the discourse seeking to circumscribe professionalism in wound nursing. Wound care nurses are convinced that they achieve better outcomes when comparing themselves to other nurses. A non-healing ulcer challenges that claim and with it the boundary work invested in advocating for the specialism [30]. In contrast to the ambiguous nature of the social ulcer, a patient’s social situation directly becomes part of the therapeutic relationship in the case of out-of-pocket payments for home care. It introduces negotiations about levels of service, liability, payment schedules, and nurse’s guilt.

Our analysis adds further evidence to the systemic care fragmentation in Austria that we already flagged in the background section. It is not that interprofessional relationships do not exist, but that they are informal and rely on networking by nurses. In practice, this means that whether a patient benefits from a treatment plan that is agreed on and consistently executed by a care collective, depends to a significant degree on the extent of the informal network cultivated by their nurse. This is an issue of the legally and culturally enshrined professional hierarchy in Austrian healthcare that does not account for the autonomy of the wound nursing specialism [11], the circumstance that home care nurses might be self-employed, thus not belonging to the same organisation as the responsible physician, and that self-employed nurses can invoice patients, but not bill the health insurance [21]. Having established well-oiled relationships with doctors and hospitals is a coping strategy in a flawed system, which, however, is sometimes reframed as marketable assets that could translate into shorter waiting times for their patients in these clinics. For wound nurses, having good relations with doctors can mean more professional autonomy as well as the prevention of unnecessary procedures that patients sometimes endure when interprofessional communication fails. Looking out for one’s patient’s interests might seem commendable in the individual case, but it contributes to sorting health outcomes along an economic gradient – one of the ‘invisible barriers’ to health access also identified by Schwarz et al. [17].

Our results illustrate that wound nurses are caught in legislative limbo. They acquire additional expertise through continued education programmes yet are not granted more prerogatives than those who only have the base qualification of registered nurse. Curricula of such training are unstandardised in training content and duration. Meanwhile, health policy planning propagates an ill-defined notion of ‘wound nurse’ for planning purposes. The wound nurse profile is at once promoted and muddled, which is not ideal for fostering role-based trust. Even though this point is clearly attributable to Austrian law, it can be read as an instance of the evolution of nursing roles in Europe, where new practices seem to precede their eventual legal codification [14].

**Fig. 3:**
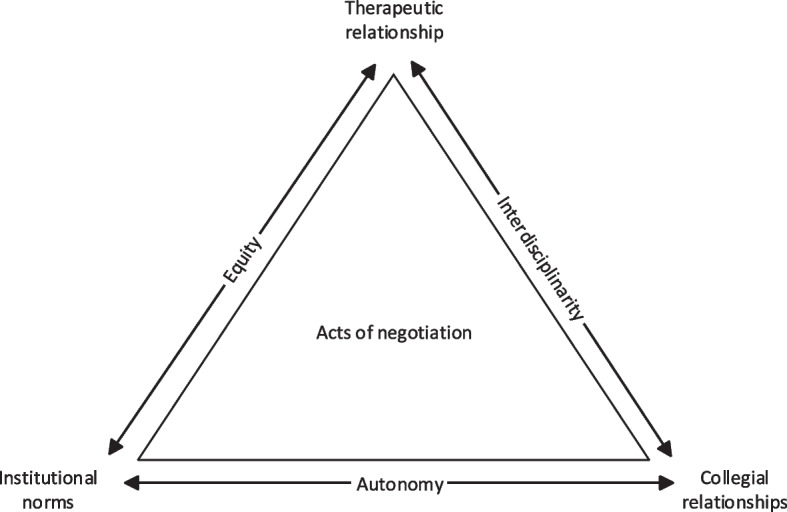
Nursing practice as acts of negotiation

Figure 3 illustrates the three themes of our analysis in a triangular shape. One common theme underpinning the three focused codes of our analysis is that ‘acts of negotiation’ are necessary to practice wound nursing. The therapeutic relationship requires complex negotiation between the self, the patient’s psychosocial situation, and norms of professionalism. Interprofessional relationships must equally be negotiated, lest a care plan falls to pieces. Finally, wound nurses must negotiate the credibility of their expertise in every professional encounter in the absence of legal norms that would standardise said expertise. We also consider bilateral connections between the themes. Role autonomy, for instance, is mediated by institutional factors as well as professional interactions. Issues of equity arise between the therapeutic relationship and the institutional distribution of resources to different care settings. Finally, the possibility of an interdisciplinary care collective sits between therapeutic and collegial relationships. It is appropriate to talk about a theory of nursing *practice*, given that situations of wound nursing are structured by proximal and distal relationships, as well as institutional dynamics that reach beyond an individual healthcare system.

## Conclusion

In this paper, we provided an analysis of the transcripts of 14 interviews, which we led with nurses who specialise in chronic wound care in Austria. Our aim was to contribute to an empirically grounded theory of nursing practice in this domain. Using constructivist grounded theory, we identified three themes that we explored in detail: 1) the therapeutic relationship, 2) collegial relationships, 3) institutional norms. We argued that they share a mutual logic, as each of them describes a situation where nurses are forced into negotiating their position as an indispensable part of their professional practice.

This study makes a novel contribution to the literature on chronic wound care because it explicitly considers the institutional context of nursing practice. It complements the scholarship that seeks to establish the evidence base of wound nursing (cf. [31]), for any care practice takes place in an institutionally patterned setting that provides specific affordances and barriers.

The study detailed care gaps in the Austrian wound care system and elaborated the mechanisms behind them. Sharpening the profile of wound nurses and allowing them to invoice statutory health insurance providers directly, are two concrete policy interventions whose potential impact ought to be evaluated in more depth. This could provide a basis for strengthening chronic care in Austria beyond wounds, and contribute to a more resilient health system in times of demographic ageing and a dwindling primary health workforce.

## Authors’ contribution 

Initial paper idea by DD. DD and RH jointly conceptualized the paper; DD conducted the interviews and data analysis; RH and DD jointly interpreted the findings and wrote the manuscript. All authors contributed to, read, and approved the final manuscript.

### Supplementary Information

**Additional file 1.**

## Data Availability

The primary data of this study consists of qualitative interview transcripts, which were exclusively carried out for the purpose of this study and have not been published elsewhere. They are not publicly available to protect interviewee confidentiality.
