# Supplementary material for: Acts of negotiation: toward a grounded theory of nursing practice in chronic wound care in Austria
Source: BMC Health Serv Res. 2023 Nov 14;23:1253. doi: 10.1186/s12913-023-10276-2 (PMC10647116; doi:10.1186/s12913-023-10276-2)
Supplement: Supplementary file 1 — Additional file 1. [file 12913_2023_10276_MOESM1_ESM.pdf]

Interview topic guide for wound nurses

Note: The version of this document used during the research process was in German and has been translated into English for documentation purposes.

Briefing

Hello and thank you for participating in this interview. Before we begin, I would like to provide you with some important information about the interview process. Your participation in this interview is entirely voluntary, and you have the right to withdraw at any time, this will not affect you or the research in any way.
This interview aims to explore your experiences and insights regarding your work in chronic wound care. The information you provide will contribute valuable insights to our study.
This interview is part of a larger research project on chronic wound care in Austria. It is a research collaboration between the University of Vienna and the Ludwig Boltzmann Research Group Senescence and healing of wounds.
Please be assured that all information shared during this interview will be treated with strict confidentiality. To ensure your privacy, we will use pseudonyms to refer to the interview. I will be the only person holding the key to link the pseudonyms with the actual interview. This means that your identity will be protected, and no other person will have access to your data directly. Your responses will be anonymized in any reports or publications resulting from this research, this means that no third party will have access to your personal information.
Please note that this interview will be recorded and, if you agree, I will also take some written notes during the interview. The recording and the notes both serve for the purpose of accurately capturing the details of our conversation. The recording will subsequently be transcribed for the analysis. The recorded data, the notes, as well as the interview transcripts will be stored securely. These materials – i.e. the raw data – will only be used for research purposes. They will not be published or shared with anyone outside our research group.
Your verbal consent to participate in this interview and acknowledgment of the recording will be assumed unless you explicitly state otherwise. If you have any questions or concerns at any point during the interview, please feel free to let me know. Your well-being during the entire interview and informed consent are our top priorities.
Thank you once again for your willingness to share your insights. Let's proceed with the interview.

Questions related to professional life in general

- To start off, tell me a bit about yourself and what you do professionally.
- How did you start working in wound care?
- How does a typical day in your work life look like?
- What do you enjoy about your work?
- What do you experience as challenging about your work?

<u>(For those who work in nurse-led clinics or home care)</u>
- How would you describe working in the clinic?
- How would you describe working in home care?

(If the interviewee worked in another medical field before)

- How would you describe your work now compared to your work in field _____?
- In your perception, are there differences in the field of wound care, compared to other medical fields?

Questions about patients and relatives

- Please describe a typical first admission with a patient.
  - Are first assessments depending on the setting (is the first admission in home care different from the clinic?)
  - What are the initial steps during the first assessment?
  - How do you educate the patient about the wound and the treatment?

- Can you describe a typical interaction with patients?
  - How do you experience the communication with the patients?
  - Are patients interested about what will happen to them?
  - Do patients share their story of wound care with you?
  - Do you set any treatment goals in wound care? What kind of goals?
  - How do you do you go about meeting these goals?
  - What would be a typical goal in wound care? Is this realistic?

(If self-employed)

- How do patients find out about you?
  - (How do they / or their relatives learn about you and your work?)
  - In what form does the initial contact happen? (patients or relatives)

- How do you document the healing process of a wound?
  - What are your thoughts on the documentation? (what works well, what could be improved, how are your records on the wound linked with the patient's other medical records?)

- Which factors are conducive to wound healing?
  - Including work of providers, patient characteristics, medication, social setting.

- To what extent are family members involved in the treatment process?

- What are your experiences of involving family members in wound care?

(If family members are involved)

- How do you include family members in wound care? (active involvement)
  - How can family involve themselves in wound care? (intrinsic involvement)
  - How can family members contribute to wound care? (their role & potentials)
  - Besides family members, do you experience other relatives, neighbors, or other caring for wound patients?

- What are your thoughts on informal care?

(I.e., care conducted by relatives, neighbors, or other informal caregivers)

Questions about other health professionals

• Besides of you, who else is typically involved in the wound care of a patient? (Professions)
• In your opinion, who else should be involved in wound care?
• How would you describe your work with other healthcare professionals?
 - How do you collaborate?
 - How would you describe your experiences? (what works well, what is challenging, and why, how could it be improved)
• How would you describe the communication flows between you and other professionals? (what works well, what is challenging, and why, how could it be improved)

Questions related to wound care in the Austrian health system

• How would you describe inpatient / outpatients / hospital / private / primary care in Austria? <u>(Formulation of question depends on the setting in which the interviewee has worked or is currently working)</u>

• How would you describe wound care in Austria?
• Based on your experiences, what could be improved / what works well in Austria's' wound care?
• What do you consider "important" in wound care?
• How would you describe "successful wound care"?
• What does "wound healing" mean to you? (Beyond wound closure)
• In your opinion, what could policymakers do for wound care in Austria?

Debriefing

Thank you for sharing your time and insights during our interview. Your contribution has been immensely valuable to our research; it provided us with a deeper understanding of the intricacies of wound care. Your perspective adds a unique layer to our study, and we appreciate your openness in sharing your experiences.
How was the interview experience for you? Your well-being and satisfaction are crucial to us, and if there are any aspects you'd like to discuss or provide feedback on, please feel free to do so. We want to ensure that the interview process is respectful and aligns with your expectations.
Is there anything you feel was left unexplored or any topics you would like to revisit? Your input can guide us in refining our research focus and ensuring that we capture all relevant aspects.
What motivated you to participate in this research? Understanding your perspective helps us appreciate the significance of your involvement and contributes to the context of your responses.
Lastly, if you have any further questions or if there are colleagues or acquaintances you think might be interested in participating in our study, please do let us know. Your network could potentially enrich our study.

We look forward to incorporating your insights into our research and contributing to a deeper understanding of wound care. Would you like to be informed about forthcoming publications? We would be pleased to stay in touch and share our research progress with you.

Thank you again for your time.
